# Sialometric and Sialochemical Analysis in Individuals With Pulp Stones

**DOI:** 10.3389/fcell.2020.00403

**Published:** 2020-06-12

**Authors:** Marilisa Carneiro Leão Gabardo, Prescila Mota de Oliveira Kublitski, Isabela Rodrigues Sette, Thaís Lauschner, Mariana Martins Juglair, Flares Baratto-Filho, João Armando Brancher, Edgard Michel-Crosato

**Affiliations:** ^1^School of Health Sciences, Universidade Positivo, Curitiba, Brazil; ^2^Department of Dentistry, Universidade de São Paulo, São Paulo, Brazil

**Keywords:** dental pulp calcification, saliva, organic chemicals, inorganic chemicals, sialometry, sialochemistry

## Abstract

The aim of this study was to analyze the saliva of patients with pulp stones, with sialometric and sialochemical tests. Eighty individuals, aged between18 and 65 years, of both sexes, were investigated. Patients were included in the pulp stone group when radiographic examination was suggestive of pulp stones in at least one permanent tooth, whereas those without this alteration were considered controls. Saliva was collected by stimulation, followed by salivary flowrate (SFR) and pH analysis tests. The organic components, such as urea (URE), glucose (GLU), total proteins (TPTs), alkaline phosphatase, creatinine (CRE), salivary amylase (SAM), and uric acid (URA), and the inorganic components, such as calcium, iron, and phosphorus, were evaluated by colorimetric techniques in an ultraviolet–visible (UV–vis) spectrophotometer. Differences among pulp stones and control groups were compared using Student’s *t*-test, with a significance level of *p* < 0.05. In both groups prevailed the female. Statistically significant differences between groups were observed for pH (*p* = 0.027), SFR (*p* = 0.002), alkaline phosphatase (*p* = 0.008), and URA (*p* = 0.005). None of the inorganic components showed significant difference (*p* > 0.05). In the analyses stratified by sex, difference between groups was observed for pH (*p* = 0.007) and URA (*p* = 0.003) in women. In conclusion, sialometric and sialochemical alterations occurred in patients with pulp stones, with significantly higher levels of pH, SFR, alkaline phosphatase, and URA.

## Introduction

Saliva is a complex biological fluid secreted continuously by salivary glands that performs numerous functions in the oral cavity, such as providing minerals to balance the process of dental demineralization and remineralization, and control of microbiota. This is possible because saliva contains numerous organic and inorganic components, which contribute to the equilibrium of the oral environment (Dawes et al., 2015). Saliva has also been described as a potential biological marker for systemic diseases (Zhang et al., 2012). The variation in salivary flowrate (SRF) and pH are related with systemic (Carramolino-Cuellar et al., 2018) and oral diseases (Foglio-Bonda et al., 2017). Diabetic patients present salivary glucose (GLU) alterations (Ladgotra et al., 2016), and higher levels of salivary urea (URE) and uric acid (URA) are commonly observed in patients with renal diseases (Alpdemir et al., 2018). Such alterations have also been observed in the most prevalent oral diseases, for example, increased total protein (TPT) levels in the presence of dental caries (Pandey et al., 2015) and periodontal disease (Shaila et al., 2013). In the past decades, numerous studies have described, standardized, and validated analyses of salivary organic and inorganic components (Teixeira et al., 2012; Malathi et al., 2013; Shaila et al., 2013; Pandey et al., 2015; Buche et al., 2017; Khozeimeh et al., 2017; Saha et al., 2017; Alpdemir et al., 2018; De et al., 2018; Jeyasree et al., 2018; Riis et al., 2018; Alshahrani et al., 2019). Owing to the detection of serum constituents, saliva has also been used as a tool for diagnosis, monitoring, and management of patients with both oral and systemic diseases (Malamud, 2011). The chief advantage of saliva as a diagnostic/prognostic tool is the ease of collection, considering the non-invasive nature of the procedure when compared to blood test (Zhang et al., 2012; Dawes et al., 2015).

Pulp stones are characterized by calcifications in the pulp chamber commonly observed in the general population, both in young and elderly patients (Satheeshkumar et al., 2013), although the prevalence is higher in the latter (Horsley et al., 2009). Its reported rates range widely, depending on the method of diagnosis, reaching 36.53% (Jannati et al., 2019), 46.9% (Ravanshad et al., 2015), and 83.3% (Hsieh et al., 2018) of frequency. Although its origin has not been fully elucidated (Goga et al., 2008), its formation results from the natural aging process or as a response to injuries, such as traumatic occlusion and orthodontic movement (Ertas et al., 2017). Genetic causes (VanDenBerghe et al., 1999) and, currently, the identification of nanoparticles and nanobacteria are pointed out as etiological factors (Zeng et al., 2011), which have also been found in kidney stones (Chen et al., 2010).

The entire process of calcification formation is reduced to the adhesion of calcium crystals to the cells, which hinders the permeability of mitochondria and the transport through their pores, which determines oxidative stress, apoptosis, and expression of osteopontin (OPN), a glycoprotein phosphorylated secreted in the extracellular bone matrix (Senger et al., 1979). Macrophages phagocytize and digest a number of crystals, but some end up aggregating into a mass containing OPN and epithelial cell debris, giving rise to the calculus nuclei. This biomolecular mechanism, described by Kohri et al. (2012), is similar to atherosclerotic calcification. Thus, these tissue changes follow the same pattern, and pulp calcifications should not be an exception (Foster et al., 2018).

In the clinical context, the presence of pulp stones can pose a challenge in endodontic treatment because they partially or fully obliterate the pulp chamber thus closing entrances to the root canals, which aggravates or fully disables thorough cleaning and shaping of the root canal system (Qualtrough and Mannocci, 2011).

Interestingly, the incidence of pulp stones is higher in patients with diabetes and kidney disease (Nakajima et al., 2013), including urolithiasis (Movahhedian et al., 2018; Gabardo et al., 2019), and coronary artery disease (Alsweed et al., 2019), possibly due to the inflammatory process common to them (Gopal et al., 2016). The association between them and certain systemic conditions is also supported by the fact that the mechanism of mineralization of pulp stones is similar to calcifications in other organs in the body (Carson, 1998). However, the exact etiology of this process remains unclear (Milcent et al., 2019). Despite the lack of a direct relationship between the salivary composition and the pulp stones, these alterations may be an oral factor indicating the presence of systemic changes (Horsley et al., 2009; Satheeshkumar et al., 2013).

Therefore, the present study evaluated the sialometric and sialochemical profile of patients with pulp stones in order to identify possible changes in composition of their saliva. The null hypothesis is that alterations in inorganic and organic components are not present in the saliva of these individuals.

## Materials and Methods

The local ethics committee of Universidade Positivo for research on humans (Reg. No. 2.805.133) approved this case–control study. Individuals who signed the form of informed consent were included.

Initially, dental records of patients (18–65 years old) with radiographs (panoramic, periapical, or bitewing), who were under treatment at the Universidade Positivo Dental Clinic, were selected. Initially, a questionnaire with nine items, adapted from a study by Bhattarai et al. (2018), was applied as a screening tool to exclude individuals with cognitive impairment, viral diseases such as HIV and hepatitis, and recent history of oral surgery (<1 month). Individuals with oral infection; salivary gland problems; history of radiotherapy in the head and neck region; those under treatment with antibiotics, antihistamines, antipsychotics, antidepressants, or anti-inflammatory drugs; dry mouth; and pregnant women were also excluded.

For the pulp stones group, patients with at least one permanent tooth with a radiograph showing pulp stones were selected. In the control group, patients with no evident pulp stone on panoramic or full-mouth radiographs were included. The period of recruitment of subjects for saliva collection was from October 2018 to June 2019.

The power observed in the sample was calculated, considering the value of α = 5% and the rejection of the null hypothesis (existence of difference between the groups), which resulted in a value of 76%.

### Pulp Stones Identification

Two trained and calibrated examiners (kappa = 0.88) preselected prospective individuals to be included in the pulp stones and control groups by analyzing radiographs from their dental records. A third examiner specialized in radiology (gold standard) reanalyzed the selected radiographs. In order to confirm the diagnosis, during the follow-up, bilateral bitewing radiographs were taken in patients with an initial finding of pulp stones. There was complete agreement between the previous and recent radiographic findings.

### Saliva Collection and Sialometric Analysis

One sample of saliva of each participant (*n* = 80) was collected, and three trained examiners performed this phase. Patients were instructed to perform proper oral hygiene and refrain from consumption of alcoholic beverages in the 12 h prior to the collection (Gopal et al., 2016). Patients were asked to chew a 1- × 0.5-cm sterile rubber band for 5 min for stimulation of salivary flow (Priya, 2017). Bottles were weighed before collection, which were reweighed after completion of the procedure to measure the SFR. The difference in weights of the empty and full bottles, in grams, is equivalent to milliliters of saliva/minute (ml/min), as per the so-called gravimetric method. Following collection of saliva, pH was immediately measured using a pocket meter with a direct electrode (Q400BD, QUIMIS, Diadema, SP, Brazil), and the samples were stored at −20°C (Chiappin et al., 2007). All samples were collected at least 1 h after the last meal.

### Sialochemical Analyses

Labtest Diagnóstica^®^ (Lagoa Santa, MG, Brazil) colorimetric kits were used for sialochemical analyses of URE, GLU, TPT, alkaline phosphatase (Alpdemir et al., 2018), creatinine (CRE), salivary amylase (SAM; Gopal et al., 2016), URA, calcium (Ca), and iron (Biscaglia et al., 2016). The Quimifos kit (Ebram Produtos Laboratoriais Ltda., São Paulo, SP, Brazil) was used for phosphorus (P) measurement. Table 1 shows the kits used with their respective wavelengths for reading and the unit of measurement of each compound.

**TABLE 1:** Salivary components analyzed, colorimetric kit used with respective wavelength, and unit of measurement.

Salivary components	Colorimetric kit	Wavelength (nm)	Unit

Organic			
URE	Urea CE (Labtest Diagnóstica S. A., Lagoa Santa, MG, Brazil)	600 (580–620)	mg/dl
GLU	Glucose Liquiform (Labtest Diagnóstica S. A., Lagoa Santa, MG, Brazil)	505 (490–520)	mg/dl
TPT	Total Protein Totais (Labtest Diagnóstica S. A., Lagoa Santa, MG, Brazil)	545 (530–55)	g/dl
ALP	Alkaline phosphatase (Labtest Diagnóstica S. A., Lagoa Santa, MG, Brazil)	590 (580–590)	U/L
CRE	Creatinine (Labtest Diagnóstica S. A., Lagoa Santa, MG, Brazil)	510 (500–540)	mg/dl
SAM	Salivary amylase (Labtest Diagnóstica S. A., Lagoa Santa, MG, Brazil)	660 (620–700)	U/dl
URA	Uric acid Liquiform (Labtest Diagnóstica S. A., Lagoa Santa, MG, Brazil)	520 (490–540)	mg/dl

**Inorganic**			

Ca	Cálcio Liquiform (Labtest Diagnóstica S. A., Lagoa Santa, MG, Brazil)	570 (550–590)	mg/dl
Fe	Ferro sérico (Labtest Diagnóstica S. A., Lagoa Santa, MG, Brazil)	560 (540–580)	μg/dl
P	Quimifos (Ebram Produtos Laboratoriais Ltda., São Paulo, SP, Brazil)	340	mg/dl

*URE, urea; GLU, glucose; TPT, total protein; ALP, alkaline phosphatase; CRE, creatinine; SAM, salivary amylase; URA, uric acid; Ca, calcium; Fe, iron; P, phosphorus.*

Biochemical procedures were performed by a single operator on ultraviolet–visible (UV–vis) spectrophotometer (Model UV, 1601, UV Visible Spectrophotometer, Shimadzu, Kyoto, Japan) using quartz cuvettes. The device was calibrated prior to each test. All colorimetric analyses were performed according to the manufacturers’ instructions.

### Statistical Analyses

Initially, a descriptive analysis of the data was performed. After the normality (Shapiro–Wilk) and homogeneity of variance (Levene) tests were done, the data were submitted for Student’s *t*-test to identify differences between the groups (pulp stones and control) and gender (male and female). Due to the sexual dimorphism observed in some salivary parameters, the analyses were also performed after stratification of the samples according to sex.

All analyses were performed using SPSS^®^ (IBM^®^ SPSS^®^ Statistics v. 25.0, SPSS Inc., Chicago, IL, United States), with a significance level of 5%.

## Results

Among the 80 patients included in this study, 43 (53.8%) presented with pulp stones and 37 (46.3%) were included as controls.

In the pulp stones group, 30 (69.8%) individuals were female, while in the control group, the number was 33 (89.2%) (*p* = 0.054).

**FIGURE 1:**
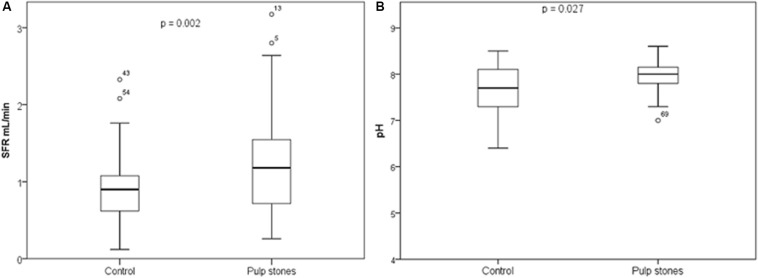
Box plot representing **(A)** salivary flowrate (SFR) (ml/min) and **(B)** salivary pH, according to pulp stones and control groups. Student’s *t*-test (*p* < 0.05).

Regarding SFR and pH, in the total sample, the means and SD were 1.09 (0.60) and 7.81 (0.45), respectively. Figure 1 shows the comparison of SFR and pH between the groups, wherein statistically significant differences were observed in both parameters, with higher mean values of both in the pulp stones (*p* = 0.002 and *p* = 0.027, respectively).

Table 2 shows the mean values of organic and inorganic components according to the groups. Statistically significant differences between groups were identified with higher mean values in the pulp stones group of ALP (*p* = 0.008) and URA (*p* = 0.005). None of the inorganic components showed significant difference (*p* > 0.05).

**TABLE 2:** Absorbance of sialochemical tests for organic and inorganic components in total subjects in the pulp stones and control groups compared (*n* = 80).

Component	Pulp stones	Control	Total	*p*-value*
URE	12.98 (10.71)	10.67 (7.70)	11.91 (9.45)	0.278
GLU	3.08 (3.19)	3.32 (2.21)	3.19 (2.77)	0.702
TPT	0.74 (0.48)	0.75 (0.38)	0.74 (0.73)	0.947
ALP	16.41 (14.97)	9.19 (7.96)	13.07 (12.69)	**0.008**
CRE	0.10 (0.92)	0.11 (0.13)	0.11 (0.11)	0.820
SAM	595.59 (248.84)	528.86 (201.99)	564.73 (229.42)	0.196
URA	3.79 (2.64)	2.17 (2.37)	3.04 (2.63)	**0.005**
Ca	7.08 (5.92)	8.13 (13.66)	7.56 (10.19)	0.647
Fe	101.42 (46.08)	88.03 (67.23)	95.23 (56.86)	0.296
P	17.31 (5.87)	15.12 (7.39)	16.30 (6.67)	0.144

*Values presented as mean (± SD). URE, urea; GLU, glucose; TPT, total protein; ALP, alkaline phosphatase; CRE, creatinine; SAM, salivary amylase; UR, uric acid; Ca, calcium; Fe, iron; P, phosphorus. ^∗^Student’s *t*-test. Bold values mean statistically significant (*p* < 0.05).*

In the comparison between sexes in the same group, female individuals presented statistically significant lower levels of P in the control group (*p* = 0.011). Similarly, in the pulp stones group, female individuals showed lower levels of URA (*p* = 0.023), SFR (*p* = 0.038), and ALP (*p* = 0.034).

In female, within the total sample, statistically significant differences were observed between the groups (pulp stones and control) for pH (*p* = 0.007) and for URA (*p* = 0.003) (Table 3).

**TABLE 3:** Results of sialometric and sialochemical tests compared between groups, stratified by sex (*n* = 80).

Variable	Male		*p*-value*	Female		*p*-value*
	Pulp stones	Control		Pulp stones	Control	
SFR	1.55 (0.82)	1.20 (0.83)	0.465	1.08 (0.58)	0.90 (0.40)	0.157
pH	7.98 (0.21)	7.75 (0.48)	0.405	7.95 (0.33)	7.63 (0.55)	**0.007**
URE	15.61 (11.96)	14.61 (10.56)	0.883	11.85 (10.13)	10.19 (7.35)	0.458
GLU	3.16 (3.08)	2.19 (0.97)	0.555	3.05 (3.29)	3.46 (2.29)	0.567
TPT	0.57 (0.22)	0.80 (0.45)	0.171	0.82 (0.54)	0.74 (0.37)	0.520
ALP	23.68 (19.40)	6.07 (4.73)	0.099	13.26 (11.61)	9.57 (8.24)	0.156
CRE	0.08 (0.04)	0.07 (0.00)	0.600	0.11 (0.11)	0.11 (0.14)	0.981
SAM	627.06 (235.41)	683.48 (148.73)	0.661	581.96 (257.11)	510.12 (201.19)	0.219
URA	3.82 (2.83)	4.68 (1.97)	0.583	3.78 (2.59)	1.87 (2.26)	**0.003**
Ca	7.41 (6.93)	15.31 (11.32)	0.105	6.93 (5.54)	7.26 (13.81)	0.903
Fe	105.65 (34.11)	80.36 (48.77)	0.257	99.59 (50.81)	88.96 (69.67)	0.495
P	17.66 (5.69)	23.80 (3.03)	0.060	17.16 (6.04)	14.07 (7.07)	0.068

*Values presented as mean (de Almeida et al., 2008). SFR, salivary flowrate; URE, urea; GLU, glucose; TPT, total proteins; ALP, alkaline phosphatase; CRE, creatinine; SAM, salivary amylase; URA, uric acid; Ca, calcium; Fe, iron; P, phosphorus. ^∗^Student’s *t*-test. Bold values mean statistically significant (*p* < 0.05).*

## Discussion

In recent years, owing to rapid advancement in research, saliva has become well recognized as a pool of biological markers. However, to the best of our knowledge, this is the first study to evaluate biochemical parameters of saliva in individuals with pulp stones. The results presented in this study rejected the null hypothesis, indicating certain salivary changes, which include alterations in pH, SFR, ALP, and URA, in patients with pulp stones.

The choice for investigating pulp stones in this research was due to the high prevalence of this alteration observed in the general population (Horsley et al., 2009; Satheeshkumar et al., 2013; Jannati et al., 2019). With an origin not elucidated (Goga et al., 2008), some authors attributed its formation to aging, injuries, orthodontic movement (Ertas et al., 2017), and genetics (VanDenBerghe et al., 1999). What is known is that there are similarities in the calcification processes (Carson, 1998; Kohri et al., 2012; Foster et al., 2018), with an exacerbation of the inflammatory process (Gopal et al., 2016). Another highlight is that the incidence of pulp stones is higher in patients with diabetes and kidney disease (Nakajima et al., 2013), including urolithiasis (Movahhedian et al., 2018; Gabardo et al., 2019), and coronary artery disease (Alsweed et al., 2019). In addition, there are evidence in the literature that female individuals are more affected (Ravanshad et al., 2015; Jannati et al., 2019), but other authors have not confirmed this information (Hsieh et al., 2018; Babu et al., 2020).

From the clinical point of view, the pulp stones make endodontic treatment extremely difficult, as they cause obliterations that cannot allow an adequate chemical–surgical preparation (Qualtrough and Mannocci, 2011).

For clinical diagnosis of the systemic diseases, blood or urine samples are widely used, but recently, saliva emerges as a useful biofluid that provides rapid and accurate information for monitoring and management of patients with both oral and systemic diseases (Malamud, 2011; Rahim et al., 2015; Babu et al., 2020). In fact, it is not new that salivary compounds are a mixture of components derived from the salivary gland and blood, and both its volume and composition may vary depending on the environmental stimuli to which the individual is subjected (Malamud, 2011) or nutrition status. Thus, just as blood or urine GLU are affected by starvation or fullness, salivary components may be altered. In this sense, it is necessary to standardize a minimum time for collection after the meal. Invariably, this time varies between 1 h (Fregoneze et al., 2013), 2 h (Panchbhai et al., 2010), or more, after meal, so here, it was standardized that collection was carried out at least 1 h after food and dental hygiene.

It is known that pH and SFR changes can be observed in individuals with systemic (Carramolino-Cuellar et al., 2018) and oral disorders (Foglio-Bonda et al., 2017). SFR is an important parameter in the maintenance of oral health, and it is known that the value is directly proportional to the amount of salivary components (Teixeira et al., 2012). The normal range of SFR is 1–3 ml/min of stimulated saliva (de Almeida et al., 2008). In this study, SFR of individuals with pulp stones was higher than those in the control group. However, this result should be interpreted with caution once the SFR was not statistical different when stratified by sex in comparison between groups. However, in the comparison between sexes, in the pulp stones, men presented statistically significant higher SFR than women.

Regarding the pH levels, the main point to be investigated is that salivary pH was higher in individuals with pulp stones in the total analysis, and when the analysis was stratified by sex, female individuals with pulp stones also presented higher pH. Statistically significant difference was not observed in male individuals, but this could be due to the lower number of men in the present sample. It is assumed that the mechanism of formation of pulp stones is similar to that of calcifications occurring in other organs in the body (Carson, 1998). A study showed that kidney stones may form due to an increase in urinary pH, and high alkalinity of urine is a strong indicator of urolithiasis (Cong et al., 2014). Interestingly, a recent meta-analysis reported an association between pulp and kidney stones (Gabardo et al., 2019). It is possible that patients with pulp stones have higher pH levels in body fluids, thereby creating an environment conducive to formation of calcifications.

Although the mean difference of URE between pulp stones and control was not statistically significantly different, this organic component in the oral cavity is degraded by the ureases produced by the microbiota, resulting in the production of ammonia, contributing to the pH elevation (Sissons et al., 2007). In addition, studies have reported increase in levels of URE in patients with renal diseases (Alpdemir et al., 2018) and halitosis (Khozeimeh et al., 2017). Further studies should be conducted to investigate the association between salivary URE and pulp stones.

Increased concentration of GLU appears to be associated with an increase in SFR (Teixeira et al., 2012). The mean GLU value (3.19 mg/dl) of the samples in the present study was similar to that reported by Alshahrani et al. (2019) (3.39–4.28 mg/dl).

Total protein was not associated with pulp stones in the present study. Salivary TPT has many protective aspects, since they are adsorbed onto the surface of the enamel, forming a pellicle that regulates the process of demineralization and remineralization, in conjunction with other components, such as Ca (Humphrey and Williamson, 2001). Increased TPT levels have been reported in individuals undergoing orthodontic treatment (Teixeira et al., 2012), patients with dental caries (Pandey et al., 2015), and periodontal disease (Shaila et al., 2013). Similarly, higher ALP levels in saliva have been reported in patients with periodontitis (De et al., 2018; Jeyasree et al., 2018).

ALP is found in cells of the periodontium, such as fibroblasts, neutrophils, and osteoblasts, and is released during their migration to the site of infection (Safkan-Seppala and Ainamo, 1992). In addition, in edentulous, osteoporotic, or osteopenic individuals, the level of salivary ALP was significantly higher when compared to the control group, suggesting that this component may be an indicator of bone metabolism disorders (Saha et al., 2017). In the present study, salivary ALP was associated with pulp stones. It is possible that ALP is higher in the pulp fluids of teeth with pulp stones. It is well known that the equilibrium of tooth demineralization and remineralization is affected by ions such as Ca and P in saliva and, consequently, by ALP. One study has reported about development of caries with significant changes in P levels (Kaur et al., 2012). In the present study, a borderline association of P in both sexes it was observed (when stratified analysis was performed for the comparison between pulp stones and control). Higher levels of P were found in patients with renal diseases (Alpdemir et al., 2018) and diabetes (Ladgotra et al., 2016). Further studies should investigate salivary P levels in patients with pulp stones. Salivary Ca was not associated with pulp stones. Previously, higher salivary levels of Ca were observed in patients with renal disease (Alpdemir et al., 2018), diabetes (Ladgotra et al., 2016), and osteoporosis and osteopenia (Saha et al., 2017). Caries-free children may also demonstrate increased salivary Ca levels (Pandey et al., 2015).

Creatinine is a common parameter evaluated in patients with renal disease, and its analysis in saliva reflected the blood levels, thus can be used as a biomarker for the diagnosis of chronic kidney disease (Lasisi et al., 2016). In individuals with halitosis, decrease in CRE levels has been reported, contrary to the levels of URE and URA (Khozeimeh et al., 2017). The present result does not support that CRE could be a biomarker for pulp stones. On the other hand, URA was associated with kidney stones in this study. A previous analysis reported that the level of URA was associated with the development of arthritis and kidney stones, due to accumulation of URA crystals (Biscaglia et al., 2016). Riis et al. (2018) evaluated salivary URA levels in healthy individuals and did not observe differences among age groups, gender, and ethnicity.

It is possible that an association between URA and pulp stones was not observed in men in the present study due to the small sample size. Previous studies conducted on salivary URA demonstrated an association of the parameter with metabolic syndrome, cardiometabolic risk factors (Soukup et al., 2012), and body mass index (Martínez et al., 2017).

Salivary amylase is an enzyme that plays an important role in digestion of starch and protein. Teixeira et al. (2012) and Alshahrani et al. (2019) reported higher levels of SAM after the installation of fixed orthodontic appliances. Studies have also reported significant increase in SAM in diabetic patients, with levels reaching 2739.48 U/dl (Malathi et al., 2013) and 1671.42 U/dl (Ladgotra et al., 2016).

A previous study reported reduction in salivary Fe levels in individuals with dental caries (Buche et al., 2017). Another study concluded that patients undergoing procedures such as peritoneal dialysis and hemodialysis had a significant reduction in salivary Fe values (Alpdemir et al., 2018). In the present study, there was no statistically significant difference in salivary Fe levels between the groups.

It is important to highlight that the present study has some limitations. The study design (convenience sample) and the sample size, as well as the lack of previous history of orthodontic treatment and previous dental and occlusal trauma could present some important limitations. In addition, saliva compounds may be altered by many factors, and there is no guarantee that all patients respected the instructions before the saliva collection. Further long-term studies, with larger sample sizes are needed to confirm and understand the associations observed here.

To summarize, high prevalence of pulp stones has been reported globally (Jannati et al., 2019) and may be associated with other systemic conditions (Nakajima et al., 2013; Alsweed et al., 2019; Gabardo et al., 2019). Additionally, sialometric and sialochemical evaluations are relevant to research in this field, to understand the etiology of diseases and discover potential salivary biomarkers. Further studies should be conducted to evaluate more biomarkers associated with pulp stones as well as elucidate the association between pulp stones and systemic conditions.

## Conclusion

Sialometric and sialochemical alterations occur in patients with pulp stones, with significantly higher levels of pH, SFR, ALP, and URA being reported in the present study.

## Data Availability Statement

The datasets generated for this study are available on request to the corresponding author.

## Ethics Statement

The studies involving human participants were reviewed and approved by the Ethics Committee of Universidade Positivo for research on humans (Reg. No. 2.805.133). The patients/participants provided their written informed consent to participate in this study.

## Author Contributions

MG contributed to study conception and design, acquisition of data, analysis and interpretation of data, and drafting of the manuscript. PK contributed to acquisition of data and drafting of the manuscript. IS, TL, and MJ contributed to acquisition of data, analysis and interpretation of data. FB-F contributed to analysis and interpretation of data, and drafting of the manuscript. JB contributed to study conception and design, acquisition of data, and drafting of the manuscript. EM-C contributed to study conception and design, and critical revision. All authors contributed to manuscript revision, reading, and approving the submitted version.

## Conflict of Interest

The authors declare that the research was conducted in the absence of any commercial or financial relationships that could be construed as a potential conflict of interest.
